# P38 Audit and reaudit of penicillin allergy documentation in patients presenting through the Emergency Department at the Royal Berkshire Hospital

**DOI:** 10.1093/jacamr/dlac004.037

**Published:** 2022-02-16

**Authors:** Aniruddh Shenoy, Afsaneh Malakouti, Shabnam Iyer

**Affiliations:** Tameside And Glossop Integrated Foundation Trust, Ashton-under-Lyne, UK; Royal Berkshire NHS Foundation Trust, Reading, UK; Royal Berkshire NHS Foundation Trust, Reading, UK

## Abstract

**Background:**

Penicillin allergy is the commonest reported drug allergy in the UK. However, it is estimated that under 10% are true allergies. Evidence shows that using second-line antibiotics is associated with worse clinical outcomes, greater healthcare costs and increased risk of antimicrobial resistance.

**Objectives:**

To evaluate the completeness of allergy history taking as per Trust guidelines on penicillin allergy classification, for patients with a positive penicillin allergy status presenting through the Emergency Department (ED) at the Royal Berkshire Hospital (RBH).

**Methods:**

In the first audit, 30 consecutive patients prescribed antibiotics in the acute medical unit (AMU) via ED at RBH were selected in April 2021. Electronic patient records were reviewed for the percentage documentation of the causative antibiotic, symptoms, time and management of reaction in those with an active penicillin allergy status. Findings were fed back to the ED team at departmental teaching. Thirty consecutive patients with recorded penicillin allergy presenting to ED were selected for re-audit in June 2021.

**Results:**

A small improvement in penicillin allergy history documentation was seen between the first audit and re-audit (Figure 1). However, 56% of patients' notes still lacked enough information to classify severity. Under 5% of patients' documented allergy history constituted absolute contraindication to penicillin-based antibiotics (Figure 2).

**Conclusions:**

Penicillin allergy documentation in patients presenting through ED at RBH remained below the standard of Trust guidance despite educating the ED team on the issue. This project has instigated an update in the Trust penicillin allergy classification guidelines and a multidisciplinary quality improvement project to identify and challenge inappropriate penicillin allergy labels.

**Figure 1.**
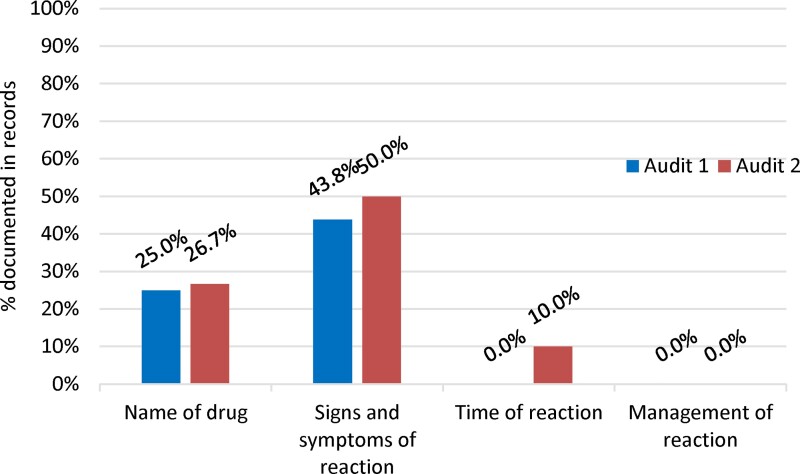
A bar chart comparing the completeness of penicillin allergy history documentation between audits 1 and 2.

**Figure 2.**
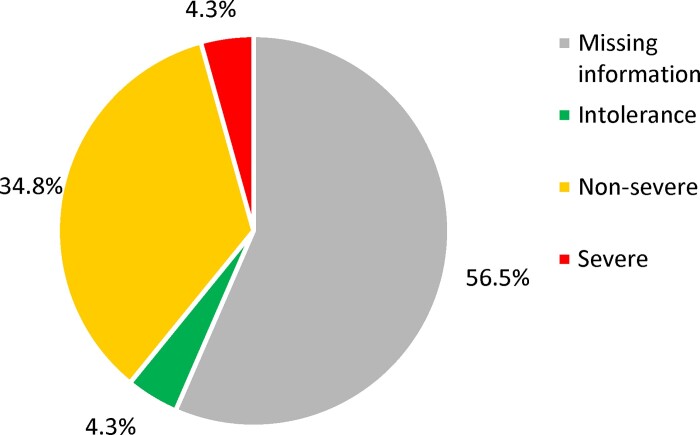
A pie chart summarizing the severity categorization by documented history in all audited patients with a recorded penicillin allergy.

